# Supplementary material for: The Arf-GAP Proteins AoGcs1 and AoGts1 Regulate Mycelial Development, Endocytosis, and Pathogenicity in Arthrobotrys oligospora
Source: J Fungi (Basel). 2022 Apr 29;8(5):463. doi: 10.3390/jof8050463 (PMC9146637; doi:10.3390/jof8050463)
Supplement: Supplementary file 1 [file jof-08-00463-s001.zip › jof-1701028-supplementary.pdf]

Supporting Information

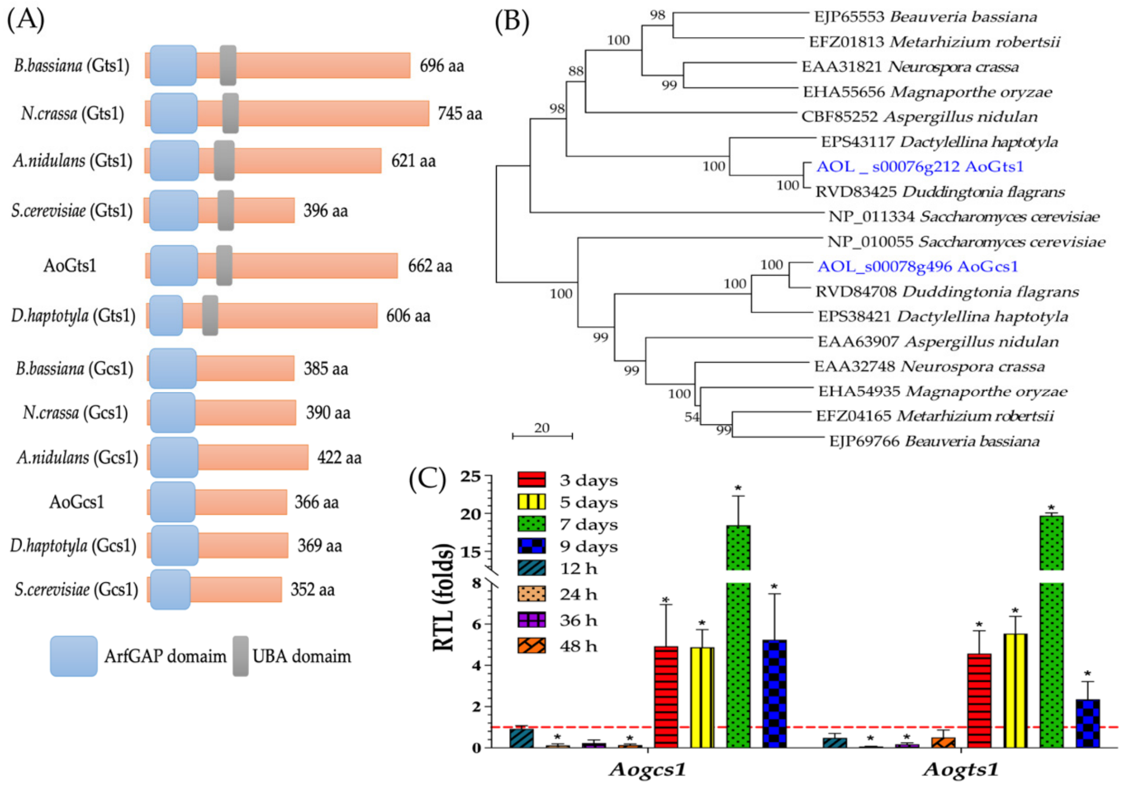

Figure S1. Multiple sequence alignment, phylogenetic analysis, and transcript-level analysis of Gcs1 and Gts1. (A) Analysis of the functional domains of Gcs1 and Gts1 proteins. (B) The phylogenetic tree of homologous Gcs1 and Gts1 from different fungi. Those orthologs of Gcs1 and Gts1 were separated into two clades. (C) Relative transcription level (RTL) of *Aogcs1* and *Aogts1* genes in the *A. oligospora* during the trap formation and conidiation stages. The red line indicates the standard (which has an RTL of 1) for the statistical analysis of the RTL of each gene in a deletion mutant compared to that in the WT strain under a given condition.

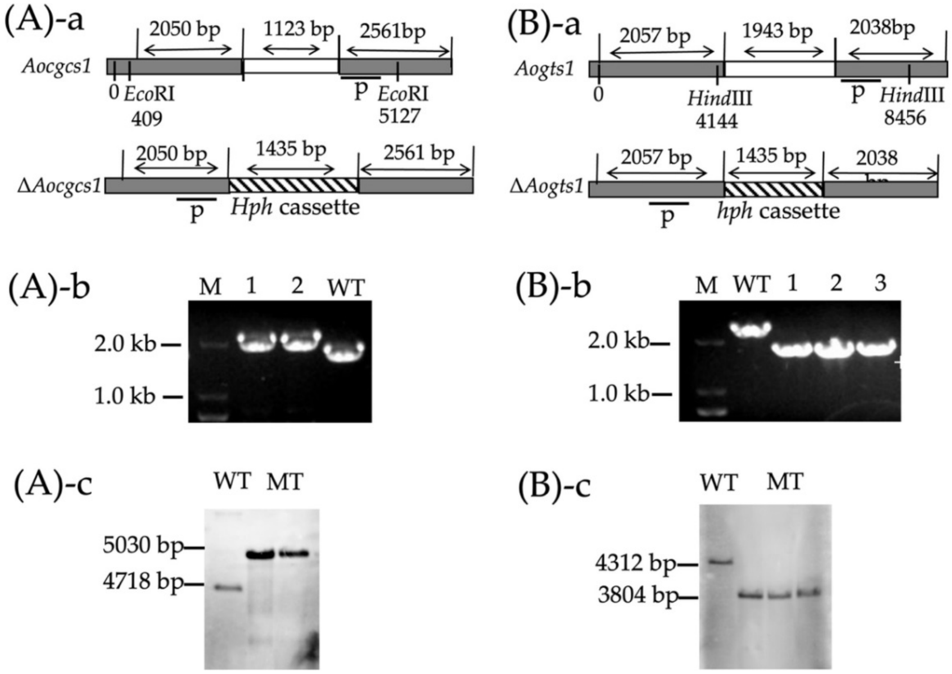

Figure S2. Knockout and verification of the genes *Aogcs1* and *Aogts1* in *A. oligospora*. (A-a and B-a) Diagrammatic sketch of homologous recombination of *AoGcs1* (A-a) and *AoGts1* (A-b). The homologous flanking sequences of the target gene, Southern blot probe, and the restriction enzyme sites are marked. (A-b and B-b) Positive transformants verified by PCR amplification. M denotes the DNA marker, and the numbers denote the positive transformants. (A-c and B-c) The Δ*AoGcs1* (A-c) and Δ*AoGts1* (B-c) mutants confirmed by Southern blots.

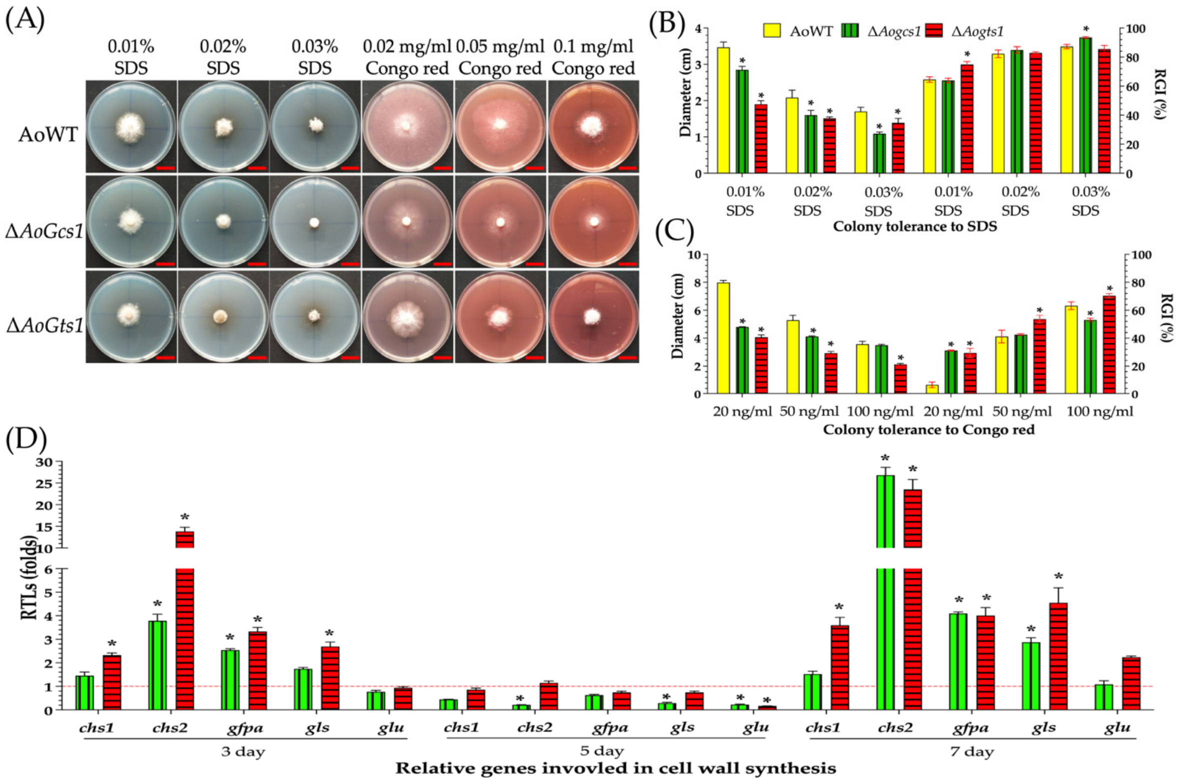

Figure S3. Comparison of stress tolerance to cell wall stress agents. (A) Colonial morphology of fungal strains under cell wall-interfering stress. Bar = 2 cm. (B-C) Colonial diameter and relative growth inhibition (RGI) of fungal colonies after being grown for 5 days on TG medium supplemented with different concentration of SDS and Congo red. (D) Transcriptional levels (RTLs) of cell wall synthesis-related genes in the mutants when compared with the WT strain at different time points. The asterisk indicates a significant difference between mutants and the WT strain ($p < 0.05$).

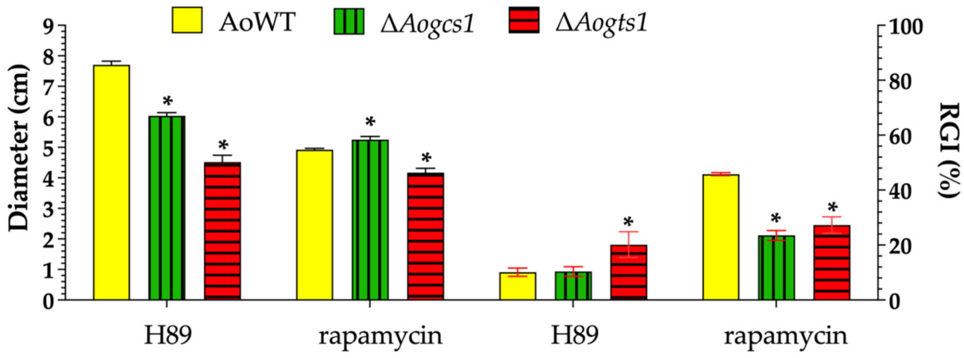

Figure S4. Colony diameters and RGI values of the WT and mutants cultured in the presence of 10 μM H-89 and 10 μM rapamycin.

Table S1. List of primers for gene disruption used in this study.

Primers	Paired sequences	Purpose
Gcs1-5F/5R	ACTGACAGCTCCAATCTCCAC/GGGGTGGT GGTAGAGCATAT	Amplify the 5′ fragment
Gcs1-3F/3R	GCTGGTGGAAAGAAGAATGA/GTGGGACTT ATGGTAATGATGC	Amplify the 3′ fragment
Gcs1YZ-F/R	ACTTGAGTGCGGAGACTGTTC/CCCAGTCAT CATTCTTCTTTCC	Verify the transformants
Gcs1P-F/R	TGATGATTGGTGATGTAGA/GAGTGGATGT AGTGAGATT	Make Southern blotting probe
Gts1-5F/5R	CCCGCCTTCTTTCTACCTCT/GACCGTTGAA TCTTGAACTGCT	Amplify the 5′ fragment
Gts1-3F/3R	AGTGGGAAGTCAGGATCGGG/CAGAACGG CACATACAATCCT	Amplify the 3′ fragment
Gts1YZ-F/R	CCTCCTTCTAAATGCCTACCT/CAAAATCCA GACTCCACCAA	Verify the transformants
Gts1P -F/R	TCGCAAAGTGGTGATGAG/ATTACCCAGTC CCAAACA	Make Southern blotting probe
Hph-F/R	GTCGGAGACAGAAGATGATATTGAAGGAG C/GTTGGAGATTTCAGTAACGTTAAGTGGAT	Amplify the *hph* cassette

Table S2. Paired primers for RT-PCR analysis of genes associated with phenotypes such as conidiation, stress resistance, serine proteases, and autophagy in *A. oligospora.*

Sporulation genes	Paired sequences (5'-3')
AOL_s00169g18 (*veA*)	AAGCTACACCCAATCAACGC/TTGCGATGCTGACGATCTTG
AOL_s00054g700 (*vosA*)	CAAACCACCCACCACCAAAT/GGATGGACAGGAGAAGGACC
AOL_s00054g811 (*velB*)	ATTCCGCAACTTCTCCCTCA/GGCATGTTTGGATTCTGGGG
AOL_s00080g63 (*abaA*)	AACTTTATGCGCCTTGTCGT/TTGGCTAGGTGGTCTGTACG
AOL_s00043g361 (*fluG*)	GATTCCAGTCCCGTGAATTC/GCTAAGGAGAGGATGGGCAT
**Cell wall biosynthesis**	**Paired sequences**
AOL_s00078g76 (*chs-1*)	GCCACTCTGCCATCTTTAGC/GCATCTTCACCCGCACCAGT
AOL_s00075g119 (c*hs-2*)	TCCCTCCAGTCCAGATGATG/TCTTGAGACCTCGACCCAAC
AOL_s00076g99 (*gfpa*)	CCATCATCGAGCACACCAAG/CCACCGAGAGTCACTGTCTT
AOL_s00083g375 (*glu*)	GTCATCCTCAAGAACGTCGC/ATATGGAAAGTTGGCCGTGC
AOL_s00054g491 (*gls*)	AGCTCTGTTCTGGTGATGCT/GATGTTTCGCCAAGGACTCC
**Antioxidant-related genes**	**Sequence (5′-3′)**
AOL_s00215g326 (*per*)	CACCATCCGCTCTGTCTTCA/GACGGCATCCTCGGTCTTGA
AOL_s00054g257 (*thr*)	TTTCGGTCAAGAGCAAAAGTG/GCTATCATTCCATTCCCCATT
AOL_s00078g209 (*trxR*)	TGTCATCGGTGGTGGAGATT/ACAAGAGAGGTAGCGGGTTC
AOL_s00076g213 (*trx*)	TTGATTTCCACGCAACCTGG/CCGAACCTGATGAAGAAGCG
AOL_s00054g13(*glr*)	CGAAAAGTCTGAATCGGGTGA/GCTCCACTTTGCCACATACATC
**Hyperosmolarity-related**	**Sequence (5′-3′)**

genes	
AOL_s00109g23 (*hog1*)	GATGTTGGAGGGAAAGCCAC/TGGGTAACGACTGGACGAATC
AOL_s00078g396 (*sho1*)	TATCAGATGCAAACGGCAGC/CTTGTAAGCACCAAACCGCT
AOL_s00081g16 (*sln1*)	TCCTACCGCCTCGAAAAGTT/CTTTCTGATGCGACACTCGG
AOL_s00112g14 (*ssk1*）	CTGATTCCTAGAGCGGGAGG/AGCAGGTAACACTCCAGGAC
AOL_s00076g216 (*msn2*)	GCGGTGATGATGACCAGTTC/TCGTCTTCGGAATCACCCAA
**Autophagy related genes**	**Sequence (5′-3′)**
AOL_s00076g234 (*atg1*)	GCCATTAGATTTGCCACCAG/CAGTTCCGTTCGTCACTCCC
AOL_s00007g534 (*atg8*)	AGCGTATCAAGCTGTCTCCC/CTCGTAGCCGAAGGTGTTTT
AOL_s00054g371 (*atg9*)	TTCTTCGGAGGACAGAGCAT/CTAGGTTTGCGTGAAAGTCG
AOL_s00215g74 (*atg13*)	AGAGGTGGAGGGTTGATTTA/GGAGTTTCCATGATGGCAGT
AOL_s00043g575 (*atg17*)	GAGATTCAGAACTCGTGGTT/AAGAAGTGAGGGTGTATTGC
**cAMP/PKA related gene**	**Sequence (5′-3′)**
AOL_s00043g480 (*pka*)	CAGGTAGTTTCGGTCGTGTG/CGTTCGTCGTTTGTGTGCT
AOL_s00078g405 (*crp*)	TTGTTGGATGCAAGAGGCC/GGGAAACCGCAAAGGAAT
AOL_s00075g84 (*cap*)	ACGAAGTCGGCCCTAATGA/GGCGGGGAAGCAGAAATAC
AOL_s00193g34 (*som1*)	CGCAGATTTGCCCTTACC/GATTTCGCCTGGACTTGGA
AOL_s00080g284 (*acy*)	GGTGAATCGTGCCGCTCGTA/TCATTGGGCTTGCTCGCTGT
AOL_s00043g12 (*fkbp12*)	GACGGCTAACGGCTCATAA/GCTTTGGGGAGAAGGACAG
AOL_s00079g187 (*rtor*)	TGCTGCTTACGAGGCTGAG/TCCCGAAGGCTGGTATGAC
AOL_s00097g622 (*mtor*)	CCGCCAGATCGCAGAATG/GTGCTAAGAACGCCAAGGAA
**Serine protease genes**	**Sequence (5′-3′)**
AOL_s00112g42	CTGGCTCTTGGCCTACTTTG/AGGAGGTTGACGGTCTCCTT
AOL_s00075g8	TTGCTACTTTACTGCCCCTTG/TCTTCAGCTTGAGTCCGGTT
AOL_s00215g702	GTCGCCGCTGACTTAACTGT/ATAATTGCTGATTCGCTGGG
AOL_s00078g136	ACACTTGCCCATTTCACTCC/GCTGGGTTTCACAACATCCT
AOL_s00054g992	TCCGCAACTTCAAGAGTGTG/CGTTGGCTTCCTCGTTAGAG
AOL_s00076g4	CCATGGTGTTGGAAGGAAAT/GTCTGTTCAGCGTACGTAGT

Table S3. The sequence similarity between AoGsc1/AoGts1 and orthologs from different fungi. The sequence similarity of orthologous AoGsc1/AoGts1 was analyzed using DNAman software.

Species	Similarity to AoGsc1 (%)	Similarity to AoGts1 (%)	Grounp
Arthrobotrys oligospora	100	100	
Duddingtonia flagrans	93.7	95.5	NT fungi
Dactylellina haptotyla	83.7	75.5	
Neurospora crassa	44.8	31.6	Other
Aspergillus nidulans	46.5	28.3	filamentous
Metarhizium robertsii	47.9	30.2	fungi
Beauveria bassiana	46.8	31.7	
Magnaporthe oryzae	46.3	29.8	
Saccharomyces cerevisiae	38.1	27.8	Yeast
